# Hydrogel‐Based Therapies for Photoaging: Current Advances and Future Perspectives

**DOI:** 10.1111/jocd.70295

**Published:** 2025-06-13

**Authors:** Qiong Gao, Chenghong Xu, Mingwei Tan, Yucheng Wang, YiDing Liu, Yifeng Wang, Yibin Fan, Xiaohua Tao, Wei Lu, Youming Huang, Yan Teng

**Affiliations:** ^1^ Center for Plastic and Reconstructive Surgery, Department of Dermatology, Zhejiang Provincial People's Hospital Affiliated People's Hospital of Hangzhou Medical College Hangzhou People's Republic of China; ^2^ Zhejiang Chinese Medical University Hangzhou People's Republic of China; ^3^ Tongde Hospital of Zhejiang Province Affiliated to Zhejiang Chinese Medical University Hangzhou People's Republic of China

**Keywords:** exosomes, hydrogels, natural extracts, photoaging, stem cells

## Abstract

**Background:**

Photoaging, caused by long‐term ultraviolet (UV) exposure, leads to wrinkles, loss of skin elasticity, pigmentation disorders, and impaired barrier function due to oxidative stress, DNA damage, and collagen breakdown. Hydrogels, with their high water content (> 90%), biocompatibility, and controlled drug release capabilities, have emerged as a powerful tool for preventing and treating skin photoaging.

**Aims:**

To review current treatment strategies and provide an updated perspective on the treatment of photoaging.

**Methods:**

We searched the PubMed and Web of Science library databases for eligible studies published within the past 5 years, categorizing and evaluating the effects of hydrogels with different carriers and active ingredients.

**Results:**

A total of 21 relevant studies were included, involving Lipid‐based Hydrogels, Hyaluronic Acid‐based Hydrogels, Chitosan‐based Hydrogels, Polyacrylonitrile‐modified *κ*‐Carrageenan‐based Hydrogels, Gellan Gum/Sodium Alginate‐based Hydrogels, Recombinant Collagen‐based Hydrogels, and Other Polymer‐based Hydrogels.

**Conclusions:**

The collected studies consistently demonstrate that hydrogel therapy is more effective than traditional treatments in reducing UV‐induced skin damage and promoting skin repair and regeneration. In addition to improving drug utilization efficiency, hydrogel carriers—particularly those incorporating hyaluronic acid—enhance skin hydration, slow aging, and accelerate wound healing, indicating promising therapeutic potential.

## 1 Introduction

Photoaging is primarily caused by UV radiation, which induces oxidative stress, DNA damage, and the degradation of collagen and elastin in the skin. Skin photoaging shows up as wrinkles, discoloration, dilated capillaries, and a dry, rough texture [1, 2], affecting both appearance and the skin's protective barrier [3, 4]. This increases the risk of inflammation and skin cancers. Traditional methods to treat the photoaging include topical creams and cosmetic surgeries [5]; however, these approaches often have their own limitations in terms of effectiveness and potential side effects. Hydrogels, with their adjustable physical and chemical properties, offer a novel and effective alternative for addressing the multifaceted issues associated with photoaging. Their ability to provide controlled drug release, improve skin hydration, and enhance the delivery of bioactive ingredients makes hydrogels a promising selection for mitigating the damage caused by UV exposure and promoting skin repair and rejuvenation.

## 2 Mechanism of UV‐Driven Skin Photoaging

Skin aging is divided into intrinsic aging and extrinsic aging, with the former being a physiological process influenced by factors such as genetics and hormones. UV radiation is the most important external factor, and skin aging caused by UV radiation is referred to as photoaging [6]. Based on wavelength, UVR can be divided into UVA (320 ~ 400 nm), UVB (280 ~ 320 nm), and UVC (100 ~ 280 nm). UVA can penetrate into the dermis and damage the extracellular matrix of the dermis, while UVB reaches the epidermis, inducing the generation of MMPs and leading to the degradation of dermal collagen fibers. UVC is filtered by the atmospheric ozone layer and therefore cannot reach the skin [7]. Besides the direct effects of UV radiation, the heat energy from sunlight may also contribute to photoaging [8]. Currently, it is believed that the pathogenesis of photoaging induced by UV radiation is mainly related to oxidative stress, DNA damage, inflammation, immune suppression, apoptosis, extracellular matrix degradation, mitochondrial dysfunction, and advanced glycation end products (AGEs) [9, 10].

### 2.1 Oxidative Stress

Cellular oxidation, coupled with an imbalance in the body's antioxidant defenses, triggers a cascade of excessive oxidative stress, culminating in the overproduction of reactive oxygen species (ROS) [11]. These highly unstable molecules wreak havoc within the cellular environment, eagerly reacting with surrounding molecules and disrupting normal function. ROS inflict direct harm on critical cellular components, including DNA, proteins, and lipids, while simultaneously driving the heightened expression of MMPs [12]. This, in turn, accelerates the degradation of collagen within the extracellular matrix, diminishing its structural integrity [13]. The cumulative effect of these intricate reactions is profound cellular dysfunction, which manifests outwardly as skin photoaging—a process characterized by increased skin laxity, the deepening of wrinkles, and a loss of youthful resilience.

### 2.2 DNA Damage

UV radiation can cause DNA double‐strand breaks, DNA strand breaks, removal or replacement of bases or base pairs, and also induce the formation of cyclobutane pyrimidine dimers and pyrimidine‐pyrimidone photoproducts, increasing the risk of skin inflammatory diseases and even malignant tumors [3, 4]. In addition, the ROS induced by UVA can cause secondary oxidative damage to DNA, exacerbating the carcinogenic effects of UVB [14, 15]. This ROS‐mediated damage amplifies the carcinogenic potential of UVB, as it exacerbates oxidative lesions like 8‐oxoguanine, further destabilizing the genome and accelerating the progression toward tumorigenesis [16]. Together, these synergistic effects of UVA and UVB underscore the complex role of UV radiation in driving skin pathology beyond mere photoaging, posing a substantial threat to long‐term skin health.

### 2.3 Inflammation and Immunosuppression

Histamine, serotonin, and kinins, along with pro‐inflammatory mediators such as TGF‐β and platelet‐derived growth factor, are the main cytokines present in skin cells that can alleviate UV‐induced damage to the dermis. These molecules contribute to tissue repair and modulate inflammatory responses, helping to restore dermal integrity following UV exposure. Moreover, TNF‐α, IL‐1, and IL‐6 indicate the immune status of photoaged skin to some extent [17]. In addition, UVB radiation induces the release of cytokines such as TNF‐α and IL‐10, further suppressing the skin's immune system [18].

### 2.4 Autophagy

Autophagy, a cellular recycling process, plays a key role in skin photoaging by mitigating UVR‐induced damage through the clearance of damaged organelles and proteins [19]. Autophagy can suppress excessive MMP expression by clearing ROS‐damaged signaling proteins or organelles that perpetuate inflammatory pathways, like NF‐κB, thereby indirectly preserving ECM integrity [20]. UVR‐induced DAMPs are normally cleared by autophagy to limit inflammatory signaling. When autophagy falters, persistent inflammation drives the release of cytokines such as IL‐1β and TNF‐α, exacerbating tissue damage and promoting a pro‐aging microenvironment [21]. Based on this, we conclude that chronic UV exposure synergizes with aging to dysregulate autophagy, thereby leading to the accumulation of cellular debris, heightened inflammation, and accelerated photoaging signs like wrinkles and collagen loss.

### 2.5 Extracellular Matrix Degradation

Matrix metalloproteinases (MMPs) can degrade various components of the extracellular matrix (ECM), especially collagen. UV radiation can upregulate the activity of MMPs, inducing the expression of MMP‐1, MMP‐3, and MMP‐9 in normal epidermis. These enzymes break down collagen fibers, leading to their progressive degradation and disorganization. As a result, the skin loses its structural integrity, strength, and elasticity, accelerating the process of photoaging and contributing to the formation of wrinkles, sagging, and other signs of premature aging [22].

### 2.6 Mitochondrial Dysfunction

UV radiation increases the likelihood of mtDNA mutations or deletions [23], affecting the function of the electron transport chain and ATP production, leading to mitochondrial dysfunction. These changes further result in increased cellular oxidative stress, elevated collagen‐degrading enzyme activity, and a vicious cycle that damages mitochondrial function, accelerating skin photoaging [24]. The clinical manifestations include profound wrinkle formation, irreversible loss of skin elasticity, and other characteristic signs of premature skin aging.

### 2.7 Ferroptosis

Ferroptosis is a novel form of iron‐dependent programmed cell death that is distinct from necrosis, autophagy, apoptosis, and other forms of cell death. UVR can induce ferroptosis in HaCaT cells, thereby exacerbating cellular aging [25]. The role of ferroptosis in skin photoaging is closely tied to its interaction with oxidative stress, a key driver of UV‐induced skin damage [26, 27]. When skin cells are exposed to UVR, it generates reactive oxygen species (ROS) that overwhelm the antioxidant defenses. This oxidative imbalance promotes lipid peroxidation, a hallmark of ferroptosis, particularly affecting polyunsaturated fatty acids in cell membranes. Iron, abundant in cells, catalyzes this process through the Fenton reaction, amplifying ROS production and pushing cells toward ferroptotic death [28]. In HaCaT cells, UVR‐induced ferroptosis disrupts normal cellular homeostasis, leading to the degradation of extracellular matrix components like collagen and elastin, which are critical for maintaining skin structure and elasticity.

### 2.8 Advanced Glycation End Products (AGEs)

Advanced glycation end products (AGEs) are formed from reducing sugars and macromolecules [29]. AGEs impair cell differentiation by disrupting signaling pathways essential for tissue repair and regeneration. Additionally, they upregulate the expression of MMPs, enzymes that degrade extracellular matrix components, thereby compromising structural integrity. AGEs also induce the production of reactive oxygen species (ROS), amplifying oxidative stress and triggering inflammatory responses. Collectively, these actions accelerate cellular aging, contributing to tissue dysfunction and age‐related pathologies, such as skin aging and chronic disease progression [30].

### 2.9 Mechanisms of Hydrogels for Skin Photoaging

**FIGURE 1:**
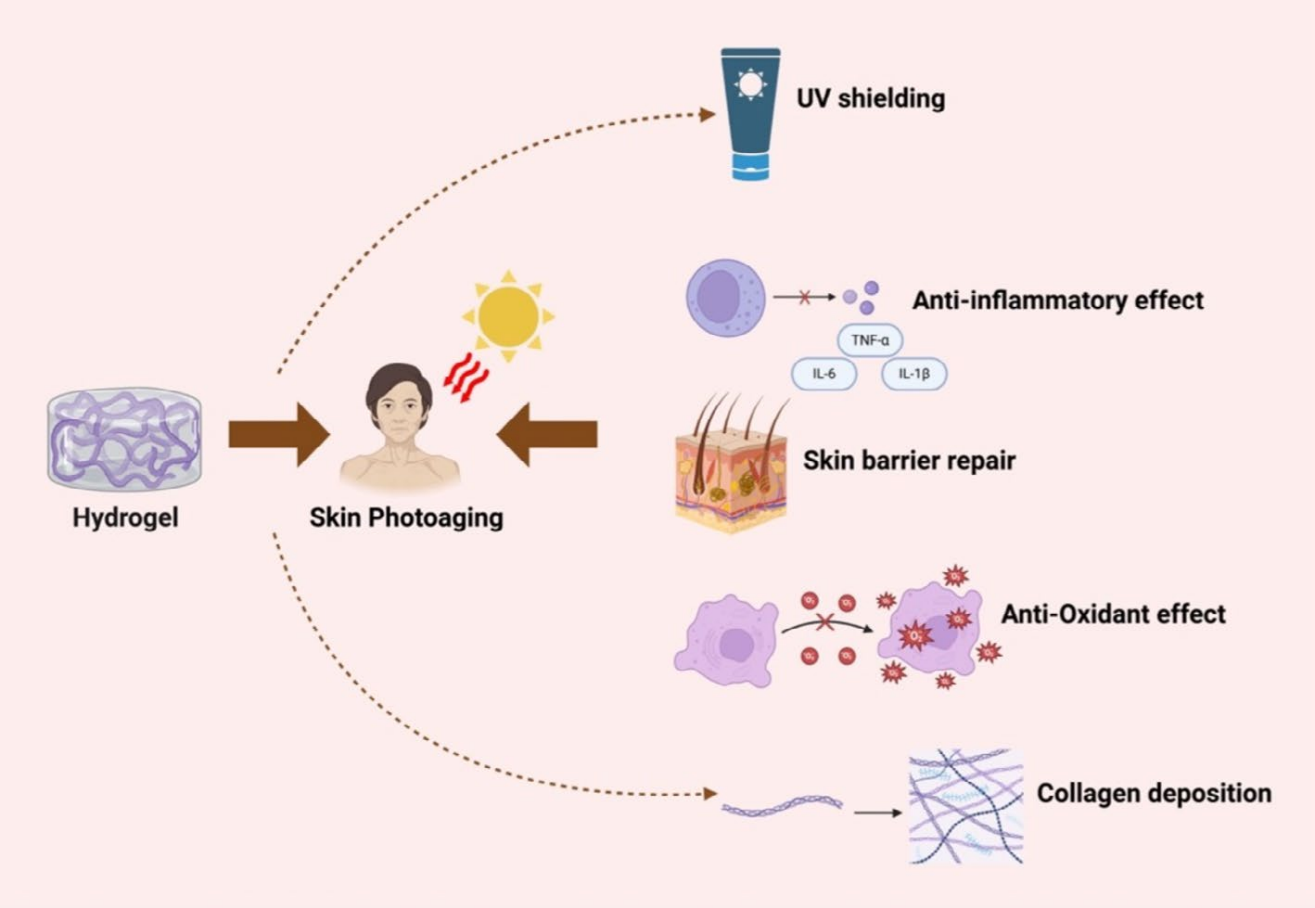
Mechanisms of hydrogels for skin photoaging.

Hydrogels are hydrated materials with a three‐dimensional crosslinked network structure that can absorb large amounts of water while maintaining shape stability. They are primarily composed of hydrophilic polymer chains, which form a network structure through chemical or physical crosslinking [31, 32]. The high water content (> 90%) of hydrogels allows them to mimic the skin's microenvironment, helping to alleviate dryness and barrier damage caused by UV radiation. On the other hand, hydrogels can load antioxidants or natural anti‐inflammatory ingredients to effectively combat photoaging. Additionally, hydrogels can incorporate growth factors (such as EGF, TGF‐β) to stimulate fibroblast proliferation and collagen synthesis, thereby mitigating skin aging. In recent years, there has been a more comprehensive understanding of the mechanisms by which hydrogels function in photoaging Figure 1.

### 2.10 UV Protection Effect

Hydrogels can effectively reduce UV penetration and slow down the photoaging process of the material by incorporating specific light shielding agents, such as UV absorbers, light stabilizers, and antioxidants. These composite systems operate via two synergistic mechanisms: primary protection through UV attenuation (via absorption and Rayleigh scattering) and secondary protection via ROS quenching. The combined effect significantly reduces incident UV radiation while simultaneously mitigating oxidative damage, thereby effectively retarding the photoaging process. This dual‐action preservation maintains hydrogel structural integrity by preventing both chain scission and undesirable crosslinking in the polymer network [33].

### 2.11 Anti‐Inflammatory Effect

Hydrogels mitigate UV‐induced inflammation by acting as biocompatible, high‐moisture carriers for anti‐inflammatory agents such as antioxidants and steroids, delivering them to damaged skin. These agents neutralize ROS and inhibit NF‐κB, reducing pro‐inflammatory cytokine expression [34]. Additionally, hydrogels' moisture‐retaining properties soothe skin barrier damage and promote inflammation resolution, aiding in photoaging prevention.

### 2.12 Antioxidant Ability

During the process of photoaging, oxidative stress is one of the main factors leading to material degradation. By incorporating natural or synthetic antioxidant molecules, such as vitamin E, glutathione, and certain phenolic compounds, into hydrogels, the generation of free radicals can be effectively reduced, slowing down the oxidation process and improving the hydrogel's anti‐aging properties [35].

### 2.13 Restoration of Skin Barrier

Owing to their high water content and flexibility, hydrogels can significantly enhance skin hydration by providing a moist environment, thereby mitigating further deterioration of barrier function [36]. Furthermore, hydrogels serve as delivery systems, encapsulating antioxidants or reparative active ingredients such as vitamin C and hyaluronic acid, effectively counteracting oxidative stress caused by photoaging while promoting epidermal cell regeneration and collagen fiber reconstruction. The physical protective layer formed by hydrogels also reduces secondary damage from external environmental factors, supporting barrier restoration.

### 2.14 MMPs Activity Suppression and Collagen Deposition

Hydrogels slow skin aging by inhibiting MMPs. Loaded with MMP inhibitors such as *Lycium barbarum* , they suppress MMP activity via localized release, inhibiting the degradation of collagen fibers in the dermis and safeguarding dermal matrix integrity [36]. Their moisturizing and barrier‐repair properties also reduce UV‐induced oxidative stress, indirectly curbing MMP overexpression and protecting against photoaging. Concurrently, by providing a moist microenvironment and encapsulating collagen‐promoting agents such as peptides or growth factors, hydrogels stimulate fibroblast activity, enhancing collagen synthesis and deposition, thereby improving the structural integrity of photoaged skin.

## 3 Applications of Hydrogels for Skin Photoaging

Hydrogels, prized for their superior hydration and biomimetic qualities, have been widely employed as coatings for medical devices over recent decades [37]. Their responsiveness to environmental stimuli such as pH, temperature, ultrasound, and light enhances their utility [38, 39, 40, 41, 42]. While UV‐protective and antioxidant drugs are widely studied, transdermal drug delivery—offering convenience, bypassing liver metabolism, and providing sustained release—has gained attention. Natural extracts, valued for low toxicity and biocompatibility, are ideal candidates, but their bioavailability is challenging to optimize. Combining them with hydrogels into topical composites enhances drug efficacy and photoaging treatment, making it a trending research focus Table 1.

**TABLE 1:** Summary of the mechanisms by which different types of hydrogels participate in photoaging repair.

Hydrogel	Compositions	Models	Biological effect	Ref
Lipid‐based hydrogels	Rice Bran Oil	Adult male Swiss mice	Enhance the stability of rice bran oil, exhibit antioxidant, anti‐inflammatory, and anti‐edema effects	Rigo et al. [43] 2015
	Tetramethylpyrazine hydrochloride	Kunming female mice, Franz diffusion pool	Exhibit strong antioxidant effects, improve skin photoaging protection, control drug release rate	Liu et al. [44] 2022
	Octyl Methoxycinnamate	Fresh pig ear skin, Hen's Egg Test‐Chorioallantoic Membrane	Enhance UV protection, trap sunscreen molecules covalently, control drug release rate	Andreani et al. [45] 2020
	Oroxylin A	HaCaT Cells, male Kunming mice	Promote transdermal drug absorption, enhance drug stability, control drug release efficiency	Zhu et al. [46] 2022
Hyaluronic acid‐based hydrogels	Exosome‐like nanovesicles derived from *Olea europaea* leaves	HaCaT Cells, HDF‐α cells, male mice	Protect against UV radiation, repair photoaged skin	Wang et al. [47] 2024
	Polylactic acid	Female ICR mice	Exhibit anti‐wrinkle effects, increase collagen production	Zhao et al. [48] 2023
	Dihydrocaffeic acid and 3‐aminophenylboronic acid	L929 Fibroblasts	Control drug release rate, protect fibroblasts	Oliveira et al. [49] 2020
	Hydroxyapatite Nanoparticles	Female BALB/c nude mice	Stimulate synthesis of collagen and elastin, enhance stiffness and gel adhesiveness	Jeong et al. [50] 2017
Chitosan‐based hydrogels	Bilberry Fruit Extract, *Vaccinium myrtillus*	HaCaT Cells	Exhibit high antioxidant activity, inhibit hyaluronidase and tyrosinase, control release, enhance bioadhesion	Sroka et al. [51] 2024
	18β‐Glycyrrhetinic Acid	HaCaT Cells, Hairless Mice	Improve skin penetration, sustain release of active components, enhance UV protection and anti‐aging effects, recover photoaged skin	Quan et al. [52] 2023
	Ascorbyl glucoside	Franz diffusion cell with porcine skin, Guinea pig skin	Promote anti‐photoaging effects, control drug release rate	Wu et al. [53] 2023
Polyacrylonitrile‐modified *κ*‐carrageenan‐based hydrogels	Sulfated galactofucan Polysaccharides and alginate oligosaccharides	HaCaT Cells, Female Kunming mice	Protect against UVB‐induced photoaging, decrease inflammation, inhibit collagen degradation	Wu et al. [54] 2024
Gellan gum/sodium alginate‐based hydrogels	Oleuropein‐rich olive leaf extract	Normal human dermal fibroblasts	Exhibit effective antioxidant activity, protect against UVA‐induced photoaging	Busto et al. [55] 2023
Recombinant collagen‐based hydrogels	Adipose‐derived mesenchymal stem cells	Nude mouse, HaCaT Cells	Reduce UV‐induced skin damage, promote skin repair and regeneration	Lin et al. [56] 2025
	Recombinant collagen	Male Kunming mice	Improve crosslinking efficiency of recombinant collagen, provide high‐performance implant material for skin regeneration	Wang et al. [57] 2024
	Cysteine‐rich thrombospondin‐1 type I repeat‐like protein	L929 Mouse Fibroblasts, C57BL/6J mice	Function as waterproof coating, enhance free radical scavenging, protect against extracellular oxidative stress	Wang et al. [58] 2022
Other polymer‐based hydrogels	Selenomethionine	Epidermal stem cells, BALB/c‐nu female nude mice	Enable sustained and controlled release of Se‐Met, reduce inflammation, remodel extracellular matrix, inhibit ferroptosis	Sun et al. [59] 2024
	Autocatalytic ceria nanoparticles	L929 Cells, HaCaT Cells, Mice	Eliminate ROS, suppress MMP production, curb collagen degradation and inflammation	Kim et al. [60] 2024
	*Lycium barbarum* Polysaccharides	Hairless mice	Reduce UVR‐induced skin damage	Neve et al. [36] 2020
	Hesperetin	Wistar rats	Protect skin from UVA‐UVB radiation damage, enhance fluidity, improve spreadability	Andrade et al. [45] 2022
	The combination of TiO_2_ with mesoporous silica	Dorsal skin of one‐week‐old pigs, HaCaT Cells, BALB/c nude mice	Alleviate skin cell death and neutrophil recruitment, resist photoaging damage from UV radiation	Lin et al. [61] 2022

### 3.1 Plant Oils and Extracts

Long‐term sun exposure generates free radicals that cause oxidative skin damage and aging. Plant oils, which are rich in bioactive compounds, have been widely studied for their natural UV‐protective properties [62]. Rigo et al. [43] developed rice bran oil‐loaded lipid‐core nanocapsules (LNCs) that attenuated UVB‐induced skin damage in mice via NF‐κB pathway inhibition, demonstrating anti‐inflammatory and antioxidant efficacy. This hydrogel enhanced active ingredient stability and skin permeability, overcoming topical delivery limitations while enabling controlled drug release [63]. Citrus fruits contain active ingredients that can be extracted at high concentrations and have been proven to possess antioxidant, anti‐inflammatory, immunoregulatory, wound‐healing, anticancer, and neuroprotective activities [64, 65, 66]. Andrade et al. [45] formulated the ammonium acryloyldimethyltaurate/VP copolymer (AAMVPC) containing 10% hesperidin, extending its duration of action. This gel prevents skin redness and swelling, significantly reduces oxidative stress, and safeguards skin from UVA‐UVB damage, preserving structural integrity. Its semi‐solid form provides suitable rheological properties and spreadability, ensuring a pleasant application experience. Studies [67] have shown that tetramethylpyrazine hydrochloride (TMPZ) not only clears reactive oxygen species (ROS), but also inhibits the excessive secretion of inflammatory factors such as interleukins (IL), cyclooxygenase‐2 (COX‐2), and tumor necrosis factor (TNF‐α) from epidermal and dermal cells [68]. Liu et al. [44] prepared a TMPZ‐loaded liposome–hydrogel (TMPZ‐LG), which enhances its antioxidant effects. The hydrogel leverages liposomes for sustained [69] and stimulus‐responsive drug release [70], improving skin adhesion and markedly enhancing bioavailability.

### 3.2 Polysaccharides and Polyphenols

Seaweed polysaccharides have various biological activities, including antioxidant, anti‐inflammatory effects, and inhibition of MMPs expression [71]. Wu et al. [54] created the FACP5 multifunctional composite hydrogel using sulfated galactofucan polysaccharides and alginate oligosaccharides as active ingredients, with polyacrylonitrile‐modified *κ*‐carrageenan as the substrate. This hydrogel demonstrates excellent biocompatibility, antioxidant, and anti‐tyrosinase activity, reducing UVB‐induced cell mortality. Its superior water retention, ease of diffusion, and strong skin adhesion make it an ideal skin protectant against photoaging. Polyphenolic compounds scavenge free radicals and delay skin aging [72]. Busto et al. [55] developed a sustainable hydrogel film by crosslinking low‐acyl gellan gum (GG) and sodium alginate (NaALG) with tartaric acid (TA), incorporating olive leaf extract (OLE) derived from agricultural food waste. This eco‐friendly formulation effectively prevented UVA‐induced photoaging through its potent free radical scavenging activity, demonstrating the potential of circular economy principles in skincare product development. Sroka et al. [51] revealed that hydrogel anti‐hyaluronidase and anti‐tyrosinase effects depend not only on incorporated extracts but also on chitosan concentration. By optimizing the formulation with 1% cranberry acetone‐water extract and 2.5% medium molecular weight (MMW) chitosan, they achieved enhanced hydrogel viscosity, leading to superior free radical scavenging and antioxidant performance.

### 3.3 Advanced Hydrogels With Bioactive Compounds

Regarding the treatment of photoaging, traditional laser therapy can promote photobiomodulation (PBM) without damaging the epidermis, stimulating protein synthesis and cell proliferation, which aids in tissue repair [73, 74]. *Lycium barbarum* fruit extract (LBP) demonstrates photoprotective and skin‐repairing properties through its bioactive components [61]. Neves et al. [36] developed a topical hydrogel with a polysaccharide‐rich LBP fraction. In a six‐week photoaging study on hairless mice, this hydrogel, alone or combined with photobiomodulation (PBM), inhibited UVR‐induced skin thickening, reduced c‐Fos, c‐Jun, and MMP‐1, ‐2, and ‐9 expression, while increasing collagen I, III, and FGF2 levels, demonstrating significant photoaging repair. Glycyrrhizic acid (18β‐glycyrrhetinic acid, GA) offers strong antioxidant and anti‐inflammatory properties [75, 76] to combat UV damage [77]. Quan et al. [52] prepared GA nanocrystals (NGAs) via high‐pressure homogenization, then combined them with amphiphilic chitosan (ACS) at a 10:1 ratio via electrostatic adsorption to form the ANGA hydrogel. This hydrogel markedly improves GA bioavailability, alleviating UV‐induced collagen fiber damage and significantly inhibiting abnormal MMP‐1 and MMP‐3 expression. *Olea europaea* leaf extract (OLEX) enhances cell vitality by inhibiting fibroblast apoptosis and exerts anti‐inflammatory and anti‐aging effects via AP‐1 and NF‐κB pathways [78, 79]. Wang et al. [47] incorporated *Olea europaea* leaf‐derived exosome‐like nanovesicles (OLELNVs) into a crosslinked hyaluronic acid (HA) and tannic acid (TA) hydrogel. The OLELNVs@HA/TA hydrogel uses HA to boost moisture for repair and TA for excellent UV absorption, effectively reducing UV‐induced damage and promoting skin regeneration, embodying a dual “defense‐repair” strategy.

### 3.4 Bioactive Hydrogels Integrated With Stem Cells and Exosomes

Recent advances in hydrogel‐based delivery systems have enabled significant progress in photoaging treatment through enhanced exosome and stem cell therapies. Exosomes, serving as key mediators of intercellular communication, demonstrate improved therapeutic efficacy when encapsulated in hydrogels due to prolonged skin retention and sustained release of bioactive molecules [80]. Similarly, plant exosome‐like nanovesicles (PELNVs) benefit from hydrogel encapsulation for precise dosage control and reduced adverse effects in natural skincare applications [81, 82, 83, 84]. While stem cells show promise for photoaging treatment through their anti‐inflammatory and antioxidant properties [85, 86, 87], challenges including poor viability and unpredictable differentiation have limited their clinical application [88, 89, 90, 91]. To address these limitations, innovative hydrogel systems have been developed, such as the microfluidic‐generated recombinant human collagen hydrogel reported by Lin et al. [56] that mechanically regulates stem cell differentiation to reduce UV‐induced wrinkles while stimulating collagen production and vascularization. Complementary to this approach, Wang et al. [58] developed a novel Ca^2+^‐self‐assembled hydrogel derived from thrombospondin‐1 protein that combines waterproof coating properties with enhanced antioxidant activity, representing a significant advancement in marine biomaterial applications for skin protection. These hydrogel‐based strategies collectively offer improved solutions for overcoming current limitations in photoaging therapies.

### 3.5 Sunscreen and Protective Coatings

Sunscreens, divided into organic and inorganic types [92], may cause irritation with prolonged use [93]. Andreani et al. [94] developed a hydrogel with SLN‐silica particles loaded with octyl methoxycinnamate (ParsolMCX), combining organic and inorganic sunscreen benefits. This hydrogel enhances UV protection through nanoparticle scattering and covalent trapping of sunscreen molecules, enabling controlled release to reduce irritation [95]. Similarly, Lin et al. [61] incorporated TiO_2_ into mesoporous silica (SBA‐15) to form a hydrogel, significantly alleviating skin cell death and neutrophil recruitment in photoaged mice, resisting UV damage via synergistic effects. Oroxylin A (OA), a flavonoid with antioxidant, anticancer, and antimicrobial properties [96, 97], was formulated by Zhu et al. [46] into OA‐nanostructured lipid carrier (OA‐NLC) hydrogels. Compared to OA solution, this hydrogel offers superior protection against UVB‐induced oxidative damage in cell models, improving transdermal absorption, drug stability, and release efficiency. Sun et al. [59] grafted Se‐Met onto UV‐responsive GelMA hydrogels via AC‐PEG‐NHS tethers. This hydrogel enhances antioxidant and UV absorption properties by inhibiting lipid peroxidation and ferroptosis while promoting GPX4 expression, significantly reducing inflammation and matrix remodeling in UV‐exposed mice.

### 3.6 Skin Fillers and Structural Support

Hydrogels provide structural support and actively participate in biological processes. Kim et al. [60] developed a hydrogel with cerium oxide nanoparticles (CeNPs), which effectively scavenge ROS, inhibit MMP production, and suppress collagen degradation and inflammation. Compared to simple fillers, this hydrogel enhances collagen matrix stability against enzymatic degradation, offering injectability and mechanical stability. It reduces skin oxidative stress, wrinkle count, epidermal thickness, and aging biomarkers while increasing collagen deposition. Wang et al. [57] reported the first tetrakis(hydroxymethyl) phosphonium chloride (THPC)‐crosslinked recombinant collagen hydrogel implant. Using THPC as a crosslinking agent, this hydrogel improves mechanical properties and stability, significantly enhancing crosslinking efficiency. It serves as a high‐performance implant for skin regeneration, improving dermal density and elasticity to combat photoaging. A hyaluronic acid‐hydroxyapatite nanocomposite hydrogel (HAc‐nanohap) was developed by Jeong et al. [50] through in situ precipitation. This composite showed superior stiffness and adhesiveness compared to pure HAc, leading to significant wrinkle reduction via stimulation of collagen and elastin synthesis, thereby reinforcing the dermal matrix.

### 3.7 Responsive Hydrogels for Controlled Release

Thermosensitive hydrogels undergo reversible phase or sol–gel transitions with temperature changes. Wu et al. [53] developed a carboxymethyl‐modified chitosan/hyaluronic acid (CMC/HA) thermosensitive hydrogel combined with ascorbyl glucoside (AA2G) liposomes. By adjusting the substitution degree, this hydrogel enhances performance, enabling gradual drug crosslinking and sustained release with temperature shifts, improving skin retention and anti‐photoaging effects. It reduces epidermal thickness, melanin deposition, and lipid oxidative damage while increasing collagen density. Hyaluronic acid (HA) hydrogel plays an important role in dermal filling. Zhao et al. [48] embedded polylactic acid (PLA) into HA using 1,4‐butanediol diglycidyl ether as a crosslinker to form an HA/PLA hydrogel. The formulation with 2 wt% PLA exhibits optimal anti‐wrinkle effects and maximal collagen production, offering high safety, injectability, and enhanced biological properties. In addition, Oliveira et al. [49] prepared a dynamic covalent hydrogel (HG) via reversible boronate ester crosslinking between HA modified with saccharide (GLU) residues and HA functionalized with 3‐aminophenylboronic acid (APBA), complexed with dihydrocaffeic acid (DHCA). The conclusion showed that at pH 7.4, this hydrogel precisely controls active substance release based on pH‐dependent dynamics, enhancing protection against UVB‐induced fibroblast death and improving drug bioavailability.

## 4 Conclusion and Future Perspective

Hydrogels have emerged as a transformative solution for combating photoaging, offering a multifunctional platform that addresses its complex pathological mechanisms. Their unique properties enable three key therapeutic actions: (1) physical UV shielding to prevent oxidative damage, (2) controlled delivery of antioxidants to neutralize free radicals, and (3) active promotion of dermal repair through collagen stimulation. This triple‐action approach makes hydrogels particularly valuable in both dermatological treatments and cosmetic applications. Advanced hydrogel systems provide more than just passive protection—they actively participate in skin rejuvenation by enhancing extracellular matrix remodeling and improving skin barrier function. Current research is driving the development of next‐generation formulations with improved bioactive stability, targeted delivery capabilities, and enhanced biocompatibility. These innovations promise to revolutionize photoaging management by combining preventive protection with restorative therapies, ultimately leading to more resilient, youthful‐looking skin.

The future of hydrogel‐based anti‐aging strategies lies in smart, responsive systems that can adapt to skin's changing needs, offering personalized protection and repair. This evolving technology represents a paradigm shift in dermatological care, moving beyond symptom management to comprehensive skin health preservation.

## Ethics Statement

The authors have nothing to report.

## Conflicts of Interest

The authors declare no conflicts of interest.

## Data Availability

The data that support the findings of this study are available from the corresponding author upon reasonable request.

## References

[1] Y. Gu , J. Han , C. Jiang , and Y. Zhang , “Biomarkers, Oxidative Stress and Autophagy in Skin Aging,” Ageing Research Reviews 59 (2020): 101036. 32105850 10.1016/j.arr.2020.101036

[2] M. Wang , P. Charareh , X. Lei , et al., “Autophagy: Multiple Mechanisms to Protect Skin From Ultraviolet Radiation‐Driven Photoaging,” Oxidative Medicine and Cellular Longevity 2019 (2019): 8135985. 31915514 10.1155/2019/8135985 PMC6930764

[3] D. A. Goukassian and B. A. Gilchrest , “The Interdependence of Skin Aging, Skin Cancer, and DNA Repair Capacity: A Novel Perspective With Therapeutic Implications,” Rejuvenation Research 7, no. 3 (2004): 175–185. 15588518 10.1089/rej.2004.7.175

[4] D. Orioli and E. Dellambra , “Epigenetic Regulation of Skin Cells in Natural Aging and Premature Aging Diseases,” Cells 7, no. 12 (2018): 268. 30545089 10.3390/cells7120268 PMC6315602

[5] Y. Teng , H. Tang , Y. Fan , and J. You , “A Bibliometric Analysis of the Top 100 Most‐Cited Articles on Skin Photoaging,” Journal of Cosmetic Dermatology 24, no. 3 (2025): e70119. 40071487 10.1111/jocd.70119 PMC11897929

[6] B. A. Gilchrest , “Photoaging,” Journal of Investigative Dermatology 133 (2013): E2–E6. 10.1038/skinbio.2013.176 23820721

[7] T. Passeron and M. Picardo , “Melasma, a Photoaging Disorder,” Pigment Cell & Melanoma Research 31, no. 4 (2018): 461–465. 29285880 10.1111/pcmr.12684

[8] C. C. E. Lan , C. S. Wu , and H. S. Yu , “Solar‐Simulated Radiation and Heat Treatment Induced Metalloproteinase‐1 Expression in Cultured Dermal Fibroblasts via Distinct Pathways: Implications on Reduction of Sun‐Associated Aging,” Journal of Dermatological Science 72, no. 3 (2013): 290–295. 24001791 10.1016/j.jdermsci.2013.07.015

[9] J. Ma , Y. Teng , Y. Huang , X. Tao , and Y. Fan , “Autophagy Plays an Essential Role in Ultraviolet Radiation‐Driven Skin Photoaging,” Frontiers in Pharmacology 13 (2022): 864331. 36278173 10.3389/fphar.2022.864331 PMC9582953

[10] I. S. Pyo , S. Yun , Y. E. Yoon , J. W. Choi , and S. J. Lee , “Mechanisms of Aging and the Preventive Effects of Resveratrol on Age‐Related Diseases,” Molecules (Basel, Switzerland) 25, no. 20 (2020): 4649. 33053864 10.3390/molecules25204649 PMC7587336

[11] T. Gao , Y. Li , X. Wang , and F. Ren , “The Melatonin‐Mitochondrial Axis: Engaging the Repercussions of Ultraviolet Radiation Photoaging on the Skin's Circadian Rhythm,” Antioxidants (Basel) 12, no. 5 (2023): 1000. 37237866 10.3390/antiox12051000 PMC10215173

[12] P. Pittayapruek , J. Meephansan , O. Prapapan , M. Komine , and M. Ohtsuki , “Role of Matrix Metalloproteinases in Photoaging and Photocarcinogenesis,” International Journal of Molecular Sciences 17, no. 6 (2016): 868. 27271600 10.3390/ijms17060868 PMC4926402

[13] A. Kammeyer and R. M. Luiten , “Oxidation Events and Skin Aging,” Ageing Research Reviews 21 (2015): 16–29. 25653189 10.1016/j.arr.2015.01.001

[14] B. R , D. D. Mc , D. T , et al., “Persistence and Tolerance of DNA Damage Induced by Chronic UVB Irradiation of the Human Genome,” Journal of Investigative Dermatology 138, no. 2 (2018): 405–412. 28951242 10.1016/j.jid.2017.08.044

[15] S. H and J. Dp , “Reactive Oxygen Species (ROS) as Pleiotropic Physiological Signalling Agents,” Nature Reviews. Molecular Cell Biology 21, no. 7 (2020): 363–383. 32231263 10.1038/s41580-020-0230-3

[16] J. Cadet , T. Douki , and J. L. Ravanat , “Oxidatively Generated Damage to Cellular DNA by UVB and UVA Radiation,” Photochemistry and Photobiology 91, no. 1 (2015): 140–155. 25327445 10.1111/php.12368

[17] P. H. Hart , M. A. Grimbaldeston , and J. J. Finlay‐Jones , “Mast Cells in UV‐B‐Induced Immunosuppression,” Journal of Photochemistry and Photobiology, B: Biology 55, no. 2–3 (2000): 81–87. 10942071 10.1016/s1011-1344(00)00032-4

[18] S. La , R. K , W. M , et al., “UVA‐1 Exposure In Vivo Leads to an IL‐6 Surge Within the Skin,” Experimental Dermatology 26, no. 9 (2017): 830–832. 28094867 10.1111/exd.13286

[19] A. Y , S. M. T , H. M , et al., “Autophagy in Healthy Aging and Disease,” Nature Aging 1, no. 8 (2021): 634–650. 34901876 10.1038/s43587-021-00098-4 PMC8659158

[20] Y. Teng , Y. Huang , D. X , et al., “The Role of Probiotics in Skin Photoaging and Related Mechanisms: A Review,” Clinical, Cosmetic and Investigational Dermatology 15 (2022): 2455–2464. 36420112 10.2147/CCID.S388954 PMC9677255

[21] R. Fan , Y. Zhang , R. Liu , et al., “Exogenous Nucleotides Improve the Skin Aging of SAMP8 Mice by Modulating Autophagy Through MAPKs and AMPK Pathways,” Nutrients 16, no. 12 (2024): 1907. 38931262 10.3390/nu16121907 PMC11206724

[22] M. Cavinato , R. Koziel , N. Romani , et al., “UVB‐Induced Senescence of Human Dermal Fibroblasts Involves Impairment of Proteasome and Enhanced Autophagic Activity,” Journals of Gerontology. Series A, Biological Sciences and Medical Sciences 72, no. 5 (2017): 632–639. 27516623 10.1093/gerona/glw150

[23] M. Berneburg , H. Plettenberg , K. Medve‐König , et al., “Induction of the Photoaging‐Associated Mitochondrial Common Deletion In Vivo in Normal Human Skin,” Journal of Investigative Dermatology 122, no. 5 (2004): 1277–1283. 15140232 10.1111/j.0022-202X.2004.22502.x

[24] R. S. Balaban , S. Nemoto , and T. Finkel , “Mitochondria, Oxidants, and Aging,” Cell 120, no. 4 (2005): 483–495. 15734681 10.1016/j.cell.2005.02.001

[25] Y. Teng , H. Tang , X. Tao , Y. Huang , and Y. Fan , “Ferrostatin 1 Ameliorates UVB‐Induced Damage of HaCaT Cells by Regulating Ferroptosis,” Experimental Dermatology 33, no. 2 (2024): e15018. 38414007 10.1111/exd.15018

[26] Y. Teng , Y. Huang , X. Tao , Y. Fan , and J. You , “Emerging Role of Ferroptosis in Ultraviolet Radiation‐Driven Skin Photoaging: A Narrative Review,” Photochemical & Photobiological Sciences 24, no. 3 (2025): 531–542. 40063311 10.1007/s43630-025-00691-1

[27] Y. Teng , H. Cui , D. Xu , et al., “Specific Knockdown of the NDUFS4 Gene Reveals Important Roles of Ferroptosis in UVB‐Induced Photoaging,” Inflammation 48, no. 1 (2024): 223–235. 38796804 10.1007/s10753-024-02057-8

[28] Y. Teng , J. He , Y. Shen , et al., “TIMP3 Deficiency Accelerates UVB‐Induced HaCaT Cell Senescence by Regulating Ferroptosis,” Photochemical & Photobiological Sciences: Official Journal of the European Photochemistry Association and the European Society for Photobiology 24, no. 3 (2025): 499–509. 40117061 10.1007/s43630-025-00701-2

[29] E. J. Lee , J. Y. Kim , and S. H. Oh , “Advanced Glycation End Products (AGEs) Promote Melanogenesis Through Receptor for AGEs,” Scientific Reports 6 (2016): 27848. 27293210 10.1038/srep27848 PMC4904211

[30] X. Xu , Y. Zheng , Y. Huang , et al., “Cathepsin D Contributes to the Accumulation of Advanced Glycation End Products During Photoaging,” Journal of Dermatological Science 90, no. 3 (2018): 263–275. 29501392 10.1016/j.jdermsci.2018.02.009

[31] M. Mattioli‐Belmonte , S. Cometa , C. Ferretti , et al., “Characterization and Cytocompatibility of an Antibiotic/Chitosan/Cyclodextrins Nanocoating on Titanium Implants,” Carbohydrate Polymers 110 (2014): 173–182. 24906744 10.1016/j.carbpol.2014.03.097

[32] R. T. C. Cleophas , J. Sjollema , H. J. Busscher , J. A. W. Kruijtzer , and R. M. J. Liskamp , “Characterization and Activity of an Immobilized Antimicrobial Peptide Containing Bactericidal PEG‐Hydrogel,” Biomacromolecules 15, no. 9 (2014): 3390–3395. 25109707 10.1021/bm500899r

[33] C. R. Afonso , R. S. Hirano , A. L. Gaspar , et al., “Biodegradable Antioxidant Chitosan Films Useful as an Anti‐Aging Skin Mask,” International Journal of Biological Macromolecules 132 (2019): 1262–1273. 30980874 10.1016/j.ijbiomac.2019.04.052

[34] A. G. Kurian , R. K. Singh , V. Sagar , J. H. Lee , and H. W. Kim , “Nanozyme‐Engineered Hydrogels for Anti‐Inflammation and Skin Regeneration,” Nano‐Micro Letters 16, no. 1 (2024): 110. 38321242 10.1007/s40820-024-01323-6 PMC10847086

[35] Z. Zhao , T. Liu , S. Zhu , et al., “Development and Evaluation Studies of Corylin Loaded Nanostructured Lipid Carriers Gel for Topical Treatment of UV‐Induced Skin Aging,” Experimental Gerontology 153 (2021): 111499. 34329721 10.1016/j.exger.2021.111499

[36] L. M. G. Neves , N. A. Parizotto , C. R. Tim , et al., “Polysaccharide‐Rich Hydrogel Formulation Combined With Photobiomodulation Repairs UV‐Induced Photodamage in Mice Skin,” Wound Repair and Regeneration 28, no. 5 (2020): 645–655. 32590890 10.1111/wrr.12826

[37] R. I. Mehta , R. I. Mehta , J. M. Choi , A. Mukherjee , and R. J. Castellani , “Hydrophilic Polymer Embolism and Associated Vasculopathy of the Lung: Prevalence in a Retrospective Autopsy Study[J],” Human Pathology 46, no. 2 (2015): 191–201. 25543660 10.1016/j.humpath.2014.09.011 PMC4552330

[38] H. Chen , L. Guo , J. Wicks , et al., “Quickly Promoting Angiogenesis by Using a DFO‐Loaded Photo‐Crosslinked Gelatin Hydrogel for Diabetic Skin Regeneration,” Journal of Materials Chemistry B 4, no. 21 (2016): 3770–3781. 32263315 10.1039/c6tb00065g

[39] D. R. Fisher , J. Fidel , and C. A. Maitz , “Direct Interstitial Treatment of Solid Tumors Using an Injectable Yttrium‐90‐Polymer Composite,” Cancer Biotherapy & Radiopharmaceuticals 35, no. 1 (2020): 1–9. 31621382 10.1089/cbr.2019.2947 PMC7044762

[40] R. Raman , T. Hua , D. Gwynne , et al., “Light‐Degradable Hydrogels as Dynamic Triggers for Gastrointestinal Applications,” Science Advances 6, no. 3 (2020): eaay0065. 32010768 10.1126/sciadv.aay0065 PMC6968934

[41] M. Rizwan , R. Yahya , A. Hassan , et al., “Erratum: pH Sensitive Hydrogels in Drug Delivery: Brief History, Properties, Swelling, and Release Mechanism, Material Selection and Applications,” Polymers 9, no. 6 (2017): 225. 30970818 10.3390/polym9040137 PMC6432076

[42] T. Koga , K. Tomimori , and N. Higashi , “Transparent, High‐Strength, and Shape Memory Hydrogels From Thermo‐Responsive Amino Acid‐Derived Vinyl Polymer Networks,” Macromolecular Rapid Communications 41, no. 7 (2020): e1900650. 32078206 10.1002/marc.201900650

[43] L. A. Rigo , C. R. da Silva , S. M. de Oliveira , et al., “Nanoencapsulation of Rice Bran Oil Increases Its Protective Effects Against UVB Radiation‐Induced Skin Injury in Mice,” European Journal of Pharmaceutics and Biopharmaceutics: Official Journal of Arbeitsgemeinschaft Fur Pharmazeutische Verfahrenstechnik e.V 93 (2015): 11–17. 25818120 10.1016/j.ejpb.2015.03.020

[44] C. Liu , Y. Xia , Y. Li , et al., “Ligustrazine as an Extract From Medicinal and Edible Plant Chuanxiong Encapsulated in Liposome‐Hydrogel Exerting Antioxidant Effect on Preventing Skin Photoaging,” Polymers 14, no. 21 (2022): 4778. 36365773 10.3390/polym14214778 PMC9655468

[45] A. T. de Araújo , L. Heimfarth , D. M. Dos Santos , et al., “Hesperetin‐Based Hydrogels Protect the Skin Against UV Radiation‐Induced Damage,” AAPS PharmSciTech 23, no. 6 (2022): 170. 35729366 10.1208/s12249-022-02323-8

[46] Z. S , Z. Z , Q. W , et al., “The Nanostructured Lipid Carrier Gel of Oroxylin A Reduced UV‐Induced Skin Oxidative Stress Damage,” Colloids and Surfaces B: Biointerfaces 216 (2022): 112578. 35636325 10.1016/j.colsurfb.2022.112578

[47] W. Z , J. Y , Y. X , et al., “ *Olea europaea* Leaf Exosome‐Like Nanovesicles Encapsulated in a Hyaluronic Acid/Tannic Acid Hydrogel Dressing With Dual “Defense‐Repair” Effects for Treating Skin Photoaging,” Materials Today Bio 26 (2024): 101103. 10.1016/j.mtbio.2024.101103 PMC11201150 38933415

[48] J. Zhao , Z. Chen , X. Li , et al., “Performance Assessment of an Injectable Hyaluronic Acid/Polylactic Acid Complex Hydrogel With Enhanced Biological Properties as a Dermal Filler,” Journal of Biomedical Materials Research, Part A 112, no. 5 (2024): 721–732. 38093473 10.1002/jbm.a.37653

[49] M. M. De Oliveira , C. V. Nakamura , and R. Auzély‐Velty , “Boronate‐Ester Crosslinked Hyaluronic Acid Hydrogels for Dihydrocaffeic Acid Delivery and Fibroblasts Protection Against UVB Irradiation,” Carbohydrate Polymers 247 (2020): 116845. 32829875 10.1016/j.carbpol.2020.116845

[50] S. H. Jeong , Y. Fan , K. H. Cheon , et al., “Hyaluronic Acid‐Hydroxyapatite Nanocomposite Hydrogels for Enhanced Biophysical and Biological Performance in a Dermal Matrix,” Journal of Biomedical Materials Research, Part A 105, no. 12 (2017): 3315–3325. 28865186 10.1002/jbm.a.36190

[51] E. Studzińska‐Sroka , M. Paczkowska‐Walendowska , C. Erdem , et al., “Anti‐Aging Properties of Chitosan‐Based Hydrogels Rich in Bilberry Fruit Extract,” Antioxidants 13, no. 1 (2024): 105. 38247529 10.3390/antiox13010105 PMC10812676

[52] W. Quan , S. Kong , S. Li , et al., “Anti‐Photoaging Effects of Nanocomposites of Amphiphilic Chitosan/18β‐Glycyrrhetinic Acid,” Molecules (Basel, Switzerland) 28, no. 11 (2023): 4362. 37298838 10.3390/molecules28114362 PMC10254817

[53] S. Wu , G. Liu , P. Shao , et al., “Transdermal Sustained Release Properties and Anti‐Photoaging Efficacy of Liposome‐Thermosensitive Hydrogel System,” Advanced Healthcare Materials 13, no. 2 (2024): 2301933. 10.1002/adhm.202301933 37607774

[54] Y. Wu , L. Geng , J. Zhang , et al., “Preparation of Multifunctional Seaweed Polysaccharides Derivatives Composite Hydrogel to Protect Ultraviolet B‐Induced Photoaging In Vitro and In Vivo,” Macromolecular Bioscience 24, no. 4 (2024): e2300292. 37985229 10.1002/mabi.202300292

[55] B. F , L. C , L. A , et al., “Oleuropein‐Rich Gellan Gum/Alginate Films as Innovative Treatments Against Photo‐Induced Skin Aging,” Molecules (Basel, Switzerland) 28, no. 11 (2023): 4352. 37298828 10.3390/molecules28114352 PMC10254495

[56] L. X , F. Am , Z. Y , et al., “Mechanically Regulated Microcarriers With Stem Cell Loading for Skin Photoaging Therapy,” Bioactive Materials 46 (2025): 448–456. 39850019 10.1016/j.bioactmat.2024.12.024 PMC11754972

[57] Q. Wang , H. Yan , L. Yao , Y. Xie , P. Liu , and J. Xiao , “A Highly Bioactive THPC‐Crosslinked Recombinant Collagen Hydrogel Implant for Aging Skin Rejuvenation,” International Journal of Biological Macromolecules 266, no. Pt 2 (2024): 131276. 38561117 10.1016/j.ijbiomac.2024.131276

[58] L. Wang , X. Zhang , P. Xu , et al., “Exploration of Sea Anemone‐Inspired High‐Performance Biomaterials With Enhanced Antioxidant Activity,” Bioactive Materials 10 (2022): 504–514. 34901563 10.1016/j.bioactmat.2021.08.021 PMC8637015

[59] J. Sun , X. Xie , Y. Song , et al., “Selenomethionine in Gelatin Methacryloyl Hydrogels: Modulating Ferroptosis to Attenuate Skin Aging,” Bioactive Materials 35 (2024): 495–516. 38404642 10.1016/j.bioactmat.2024.02.013 PMC10885793

[60] Y. E. Kim , P. Im , S. W. Choi , and J. Kim , “Autocatalytic Ceria Nanoparticle‐Embedded Tilapia Collagen Hydrogels as Enhanced Antioxidative and Long‐Lasting Dermal Fillers for Photoaged Skin,” Nano Letters 25, no. 1 (2025): 212–221. 39718381 10.1021/acs.nanolett.4c04797

[61] Y. C. Lin , Y. P. Fang , C. F. Hung , et al., “Multifunctional TiO2/SBA‐15 Mesoporous Silica Hybrids Loaded With Organic Sunscreens for Skin Application: The Role in Photoprotection and Pollutant Adsorption With Reduced Sunscreen Permeation,” Colloids and Surfaces B: Biointerfaces 202 (2021): 111658. 33677134 10.1016/j.colsurfb.2021.111658

[62] Y. Tülüce , H. Ozkol , and I. Koyuncu , “Photoprotective Effect of Flax Seed Oil (*Linum usitatissimum* L.) Against Ultraviolet C‐Induced Apoptosis and Oxidative Stress in Rats,” Toxicology and Industrial Health 28, no. 2 (2012): 99–107. 21665902 10.1177/0748233711407239

[63] M. J. Alonso , “Nanomedicines for Overcoming Biological Barriers,” Biomedicine & Pharmacotherapy 58, no. 3 (2004): 168–172. 15082339 10.1016/j.biopha.2004.01.007

[64] A. Samie , R. Sedaghat , T. Baluchnejadmojarad , and M. Roghani , “Hesperetin, a Citrus Flavonoid, Attenuates Testicular Damage in Diabetic Rats via Inhibition of Oxidative Stress, Inflammation, and Apoptosis,” Life Sciences 210 (2018): 132–139. 30179627 10.1016/j.lfs.2018.08.074

[65] M. Aswar , P. Kute , S. Mahajan , U. Mahajan , G. Nerurkar , and U. Aswar , “Protective Effect of Hesperetin in Rat Model of Partial Sciatic Nerve Ligation Induced Painful Neuropathic Pain: An Evidence of Anti‐Inflammatory and Anti‐Oxidative Activity,” Pharmacology, Biochemistry and Behavior 124 (2014): 101–107. 24871567 10.1016/j.pbb.2014.05.013

[66] L. N. Bodduluru , E. R. Kasala , C. C. Barua , K. C. Karnam , V. Dahiya , and M. Ellutla , “Antiproliferative and Antioxidant Potential of Hesperetin Against Benzo(a)pyrene‐Induced Lung Carcinogenesis in Swiss Albino Mice,” Chemico‐Biological Interactions 242 (2015): 345–352. 26546711 10.1016/j.cbi.2015.10.020

[67] C. Y. Zheng , W. Xiao , M. X. Zhu , X. J. Pan , Z. H. Yang , and S. Y. Zhou , “Inhibition of Cyclooxygenase‐2 by Tetramethylpyrazine and Its Effects on A549 Cell Invasion and Metastasis,” International Journal of Oncology 40, no. 6 (2012): 2029–2037. 22344367 10.3892/ijo.2012.1375

[68] L. Y. Chiu , N. L. Wu , C. F. Hung , P. Bai , Y. S. Dai , and W. W. Lin , “PARP‐1 Involves in UVB‐Induced Inflammatory Response in Keratinocytes and Skin Injury via Regulation of ROS‐Dependent EGFR Transactivation and p38 Signaling,” FASEB Journal: Official Publication of the Federation of American Societies for Experimental Biology 35, no. 3 (2021): e21393. 33570794 10.1096/fj.202002285RR

[69] B. Almeida , O. K. Nag , K. E. Rogers , and J. B. Delehanty , “Recent Progress in Bioconjugation Strategies for Liposome‐Mediated Drug Delivery,” Molecules (Basel, Switzerland) 25, no. 23 (2020): 5672. 33271886 10.3390/molecules25235672 PMC7730700

[70] C. Tan , J. Wang , and B. Sun , “Biopolymer‐Liposome Hybrid Systems for Controlled Delivery of Bioactive Compounds: Recent Advances,” Biotechnology Advances 48 (2021): 107727. 33677025 10.1016/j.biotechadv.2021.107727

[71] L. Wang , T. U. Jayawardena , Y. S. Kim , et al., “Anti‐Melanogenesis and Anti‐Photoaging Effects of the Sulfated Polysaccharides Isolated From the Brown Seaweed Padina Boryana,” Polymers 15, no. 16 (2023): 3382. 37631439 10.3390/polym15163382 PMC10459840

[72] M. Działo , J. Mierziak , U. Korzun , M. Preisner , J. Szopa , and A. Kulma , “The Potential of Plant Phenolics in Prevention and Therapy of Skin Disorders,” International Journal of Molecular Sciences 17, no. 2 (2016): 160. 26901191 10.3390/ijms17020160 PMC4783894

[73] M. Esmaeelinejad and M. Bayat , “Effect of Low‐Level Laser Therapy on the Release of Interleukin‐6 and Basic Fibroblast Growth Factor From Cultured Human Skin Fibroblasts in Normal and High Glucose Mediums,” Journal of Cosmetic and Laser Therapy: Official Publication of the European Society for Laser Dermatology 15, no. 6 (2013): 310–317. 23656570 10.3109/14764172.2013.803366

[74] U. Kartal , E. Koptagel , H. E. Bulut , and H. Erdogan , “Protective Effect of Basic Fibroblast Growth Factor on Laser Induced Retinopathy,” International Journal of Ophthalmology 6, no. 6 (2013): 744–751. 24392319 10.3980/j.issn.2222-3959.2013.06.02 PMC3874510

[75] B. Chen , D. Zhu , C. Xie , et al., “18β‐Glycyrrhetinic Acid Inhibits IL‐1β‐Induced Inflammatory Response in Mouse Chondrocytes and Prevents Osteoarthritic Progression by Activating Nrf2,” Food & Function 12, no. 18 (2021): 8399–8410. 34369548 10.1039/d1fo01379c

[76] Y. K. Sun , Y. F. Zhang , L. Xie , et al., “Progress in the Treatment of Drug‐Induced Liver Injury With Natural Products,” Pharmacological Research 183 (2022): 106361. 35882295 10.1016/j.phrs.2022.106361

[77] S. Z. Kong , H. M. Chen , X. T. Yu , et al., “The Protective Effect of 18β‐Glycyrrhetinic Acid Against UV Irradiation Induced Photoaging in Mice,” Experimental Gerontology 61 (2015): 147–155. 25498537 10.1016/j.exger.2014.12.008

[78] T. K. Lin , L. Zhong , and J. L. Santiago , “Anti‐Inflammatory and Skin Barrier Repair Effects of Topical Application of Some Plant Oils,” International Journal of Molecular Sciences 19, no. 1 (2017): 70. 29280987 10.3390/ijms19010070 PMC5796020

[79] P. Machała , O. Liudvytska , A. Kicel , A. Dziedzic , M. A. Olszewska , and H. M. Żbikowska , “Valorization of the Photo‐Protective Potential of the Phytochemically Standardized Olive (*Olea europaea* L.) Leaf Extract in UVA‐Irradiated Human Skin Fibroblasts,” Molecules (Basel, Switzerland) 27, no. 16 (2022): 5144. 36014384 10.3390/molecules27165144 PMC9415354

[80] R. Wang , X. Wang , Y. Zhan , et al., “A Dual Network Hydrogel Sunscreen Based on Poly‐γ‐Glutamic Acid/Tannic Acid Demonstrates Excellent Anti‐UV, Self‐Recovery, and Skin‐Integration Capacities,” ACS Applied Materials & Interfaces 11, no. 41 (2019): 37502–37512. 31544451 10.1021/acsami.9b14538

[81] M. Colombo , G. Raposo , and C. Théry , “Biogenesis, Secretion, and Intercellular Interactions of Exosomes and Other Extracellular Vesicles,” Annual Review of Cell and Developmental Biology 30 (2014): 255–289. 10.1146/annurev-cellbio-101512-122326 25288114

[82] H. A. Dad , T. W. Gu , A. Q. Zhu , L. Q. Huang , and L. H. Peng , “Plant Exosome‐Like Nanovesicles: Emerging Therapeutics and Drug Delivery Nanoplatforms,” Molecular Therapy: The Journal of the American Society of Gene Therapy 29, no. 1 (2021): 13–31. 33278566 10.1016/j.ymthe.2020.11.030 PMC7791080

[83] O. Urzì , S. Raimondo , and R. Alessandro , “Extracellular Vesicles From Plants: Current Knowledge and Open Questions,” International Journal of Molecular Sciences 22, no. 10 (2021): 5366. 34065193 10.3390/ijms22105366 PMC8160738

[84] T. W. Gu , M. Z. Wang , J. Niu , Y. Chu , K.‐R. Guo , and L.‐H. Peng , “Outer Membrane Vesicles Derived From *E. coli* as Novel Vehicles for Transdermal and Tumor Targeting Delivery,” Nanoscale 12, no. 36 (2020): 18965–18977. 32914815 10.1039/d0nr03698f

[85] S. He and N. E. Sharpless , “Senescence in Health and Disease,” Cell 169, no. 6 (2017): 1000–1011. 28575665 10.1016/j.cell.2017.05.015 PMC5643029

[86] C. López‐Otín , M. A. Blasco , L. Partridge , M. Serrano , and G. Kroemer , “The Hallmarks of Aging,” Cell 153, no. 6 (2013): 1194–1217. 23746838 10.1016/j.cell.2013.05.039 PMC3836174

[87] S. J. Morrison and A. C. Spradling , “Stem Cells and Niches: Mechanisms That Promote Stem Cell Maintenance Throughout Life,” Cell 132, no. 4 (2008): 598–611. 18295578 10.1016/j.cell.2008.01.038 PMC4505728

[88] R. J. Brentjens , M. L. Davila , I. Riviere , et al., “CD19‐Targeted T Cells Rapidly Induce Molecular Remissions in Adults With Chemotherapy‐Refractory Acute Lymphoblastic Leukemia,” Science Translational Medicine 5, no. 177 (2013): 177ra38. 10.1126/scitranslmed.3005930 PMC3742551 23515080

[89] J. Chen , Y. Li , T. S. Yu , et al., “A Restricted Cell Population Propagates Glioblastoma Growth After Chemotherapy,” Nature 488, no. 7412 (2012): 522–526. 22854781 10.1038/nature11287 PMC3427400

[90] M. Nie , G. Chen , C. Zhao , et al., “Bio‐Inspired Adhesive Porous Particles With Human MSCs Encapsulation for Systemic Lupus Erythematosus Treatment,” Bioactive Materials 6, no. 1 (2021): 84–90. 32817916 10.1016/j.bioactmat.2020.07.018 PMC7419256

[91] M. Nie , B. Kong , G. Chen , Y. Xie , Y. Zhao , and L. Sun , “MSCs‐Laden Injectable Self‐Healing Hydrogel for Systemic Sclerosis Treatment,” Bioactive Materials 17 (2022): 369–378. 35386467 10.1016/j.bioactmat.2022.01.006 PMC8964965

[92] M. Krause , A. Klit , M. Blomberg Jensen , et al., “Sunscreens: Are They Beneficial for Health? An Overview of Endocrine Disrupting Properties of UV‐Filters,” International Journal of Andrology 35, no. 3 (2012): 424–436. 22612478 10.1111/j.1365-2605.2012.01280.x

[93] S. Albrecht , S. Jung , R. Müller , et al., “Skin Type Differences in Solar‐Simulated Radiation‐Induced Oxidative Stress,” British Journal of Dermatology 180, no. 3 (2019): 597–603. 30176057 10.1111/bjd.17129

[94] T. Andreani , J. Dias‐Ferreira , J. F. Fangueiro , et al., “Formulating Octyl Methoxycinnamate in Hybrid Lipid‐Silica Nanoparticles: An Innovative Approach for UV Skin Protection,” Heliyon 6, no. 5 (2020): e03831. 32395645 10.1016/j.heliyon.2020.e03831 PMC7205751

[95] Q. Xia , A. Saupe , R. H. Müller , and E. B. Souto , “Nanostructured Lipid Carriers as Novel Carrier for Sunscreen Formulations,” International Journal of Cosmetic Science 29, no. 6 (2007): 473–482. 18489386 10.1111/j.1468-2494.2007.00410.x

[96] J. Ma , S. Li , L. Zhu , et al., “Baicalein Protects Human Vitiligo Melanocytes From Oxidative Stress Through Activation of NF‐E2‐Related factor2 (Nrf2) Signaling Pathway,” Free Radical Biology & Medicine 129 (2018): 492–503. 30342186 10.1016/j.freeradbiomed.2018.10.421

[97] J. K. Seok , J. Y. Kwak , G. W. Choi , et al., “Scutellaria Radix Extract as a Natural UV Protectant for Human Skin,” Phytotherapy Research: PTR 30, no. 3 (2016): 374–379. 26620130 10.1002/ptr.5534

